# Point-of-Care Ultrasound Assessment of Ventriculoperitoneal Shunt Patency: A Case Report

**DOI:** 10.7759/cureus.110412

**Published:** 2026-06-07

**Authors:** Joseph A White, Guillermo Izquierdo-Pretel, Elham Shams

**Affiliations:** 1 Internal Medicine, Florida International University, Herbert Wertheim College of Medicine, Miami, USA; 2 Medicine, Jackson Memorial Hospital, Miami, USA

**Keywords:** bedside ultrasound, doppler ultrasound, emergency medicine ultrasound, hydrocephalus, neurosurgical devices, noninvasive diagnostic imaging, point-of-care ultrasound (pocus), shunt malfunction, shunt patency assessment, ventriculoperitoneal shunt

## Abstract

Hydrocephalus caused by obstruction of cerebrospinal fluid (CSF) flow can be life-threatening if not promptly recognized and treated. Ventriculoperitoneal (VP) shunt placement remains one of the most common therapeutic interventions; however, shunt malfunction is a frequent and potentially serious complication. Diagnosis can be challenging due to nonspecific clinical presentations and reliance on imaging modalities that may be time-consuming or limited in availability. Point-of-care ultrasound (POCUS) has emerged as a rapid, bedside diagnostic adjunct with growing clinical utility. We present the case of a 26-year-old man with a complex neurosurgical history who presented with fever, altered mental status, and concern for VP shunt malfunction. Pulse-wave Doppler POCUS was used to assess shunt patency at multiple points along the shunt pathway, demonstrating preserved CSF flow, suggesting the absence of complete mechanical obstruction. This case supports the expanding role of POCUS as a noninvasive, readily available tool for rapid VP shunt assessment.

## Introduction

Hydrocephalus results from impaired circulation or absorption of cerebrospinal fluid (CSF), leading to ventricular dilation and increased intracranial pressure with risk of permanent neurologic injury. One of the most widely used treatments is placement of a ventriculoperitoneal (VP) shunt, which diverts excess CSF from the ventricles to the peritoneal cavity [1]. Despite its effectiveness, VP shunt malfunction is common and may present with nonspecific symptoms, such as fever, headache, nausea, vomiting, or altered mental status, making timely diagnosis challenging.

Standard diagnostic modalities for suspected shunt malfunction include computed tomography or magnetic resonance imaging of the brain, radionuclide shuntography, plain radiographic shunt series, and contrast-enhanced shunt studies [1,2]. While effective, these modalities may be limited by availability, radiation exposure, procedural risk, or diagnostic delay. Sonographic techniques for evaluating shunt patency were first described several decades ago as a noninvasive alternative [2]. More recent studies have demonstrated the feasibility of Doppler-based ultrasound and advanced microvascular imaging techniques to assess CSF flow within VP shunts, supporting a potential bedside role for point-of-care ultrasound (POCUS) in suspected shunt malfunction [1,3,4]. In this report, "shunt patency" refers to Doppler-detectable CSF flow, whereas "functional shunt performance" refers to effective CSF diversion and intracranial pressure regulation. Importantly, mechanical shunt patency does not necessarily equate to effective CSF diversion or normal intracranial pressure dynamics. Despite increasing interest in ultrasound-based assessment, the evidence base remains limited and standardized protocols are lacking. This report aims to illustrate the complementary role of Doppler-based POCUS in assessing VP shunt patency while highlighting its limitations in complex intracranial physiology. Available sonographic techniques include B-mode visualization, color Doppler imaging, and pulse-wave Doppler assessment of flow dynamics.

## Case presentation

A 26-year-old man, with a past medical history significant for a motorcycle accident complicated by subdural hematoma status-post left decompressive hemicraniectomy and hydrocephalus status-post right frontal VP shunt placement one week before admission, was transferred from a long-term rehabilitation facility to the emergency department. He presented with fever, altered mental status, and nuchal rigidity concerning for central nervous system infection and possible VP shunt malfunction.

His surgical history included left decompressive hemicraniectomy and right frontal VP shunt placement. Home medications included levetiracetam 1500 mg twice daily, amantadine 100 mg twice daily, gabapentin 300 mg three times daily, and famotidine 20 mg twice daily. He had no known drug allergies. Family and social history were unobtainable.

Examinations

On admission, vital signs included temperature of 37.0°C, heart rate of 101 beats per minute, blood pressure of 124/83 mmHg, respiratory rate of 20 breaths per minute, and oxygen saturation of 100% on room air. On general examination, the patient appeared thin, alert but unresponsive, and in no acute distress. Head examination was notable for a left-sided hemicraniectomy defect and a right frontal VP shunt. Neurologic examination demonstrated altered mental status (defined as impaired responsiveness with spontaneous eye opening but no command following), fixed medial gaze, minimal response to painful stimuli, nuchal rigidity, and a positive Brudzinski sign.

Investigations

Laboratory Studies

Laboratory studies were notable for 1.8 mmol/L lactic acid (reference range: 0.7-2.1 mmol/L), 7.0 mg/dL C-reactive protein (reference range: <5.0 mg/L), and 40 mm/h erythrocyte sedimentation rate (reference range: 0-20 mm/h).

Blood cultures were obtained, and the patient was empirically treated with intravenous vancomycin, ampicillin, and ceftriaxone. Blood cultures ultimately showed no growth, and ampicillin was discontinued. A 14-day course of vancomycin and ceftriaxone was completed. Lumbar puncture and CSF analysis were not performed due to concern for elevated intracranial pressure and risk of herniation.

Imaging (Timeline-Based)

**Figure 1:**
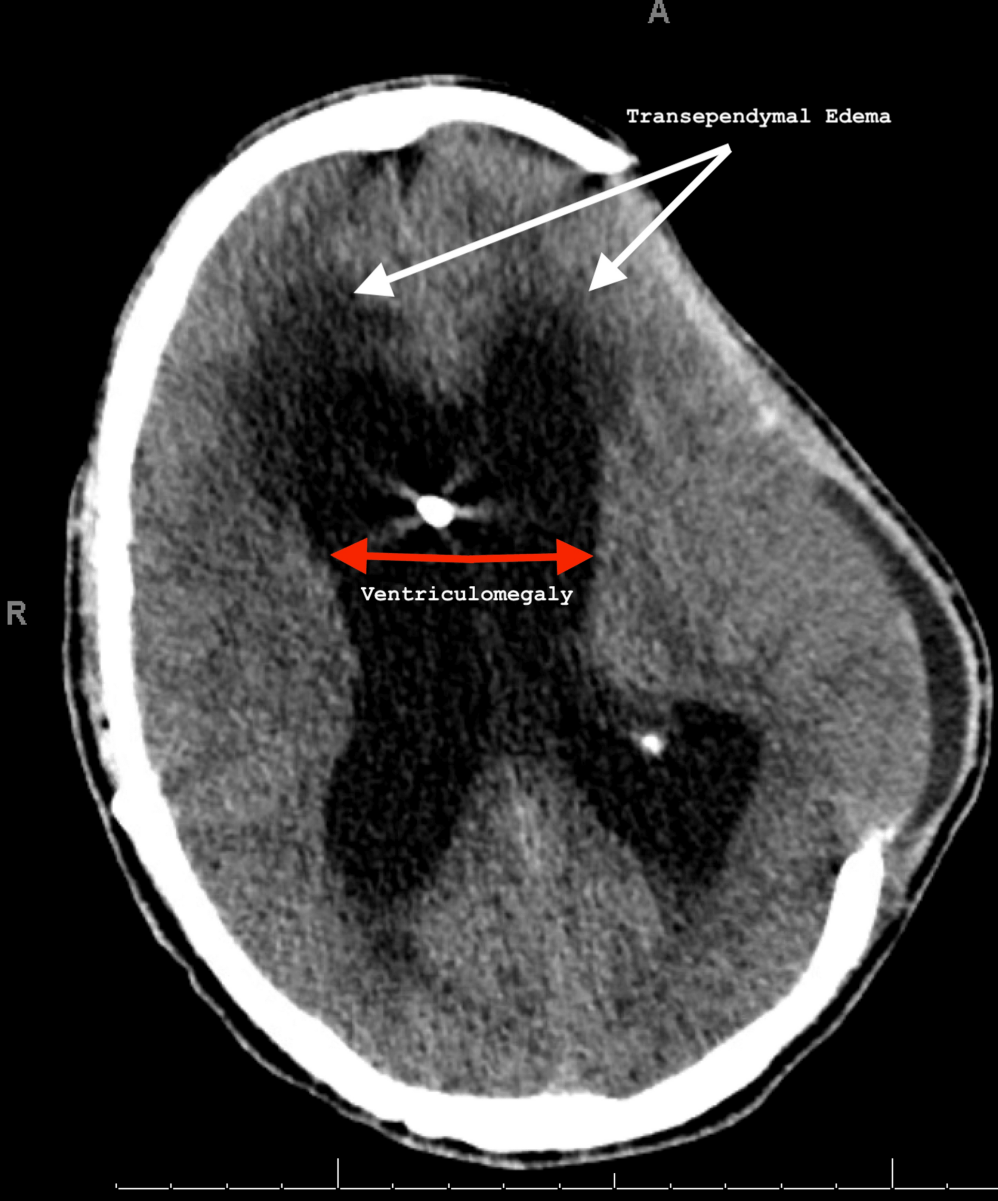
Axial contrast-enhanced CT brain demonstrating severe ventriculomegaly with enlargement of the lateral ventricles (red arrows) and surrounding periventricular hypodensity consistent with transependymal cerebrospinal fluid flow/edema (white arrows), concerning for ventriculoperitoneal shunt dysfunction. CT: computed tomography

**Figure 2:**
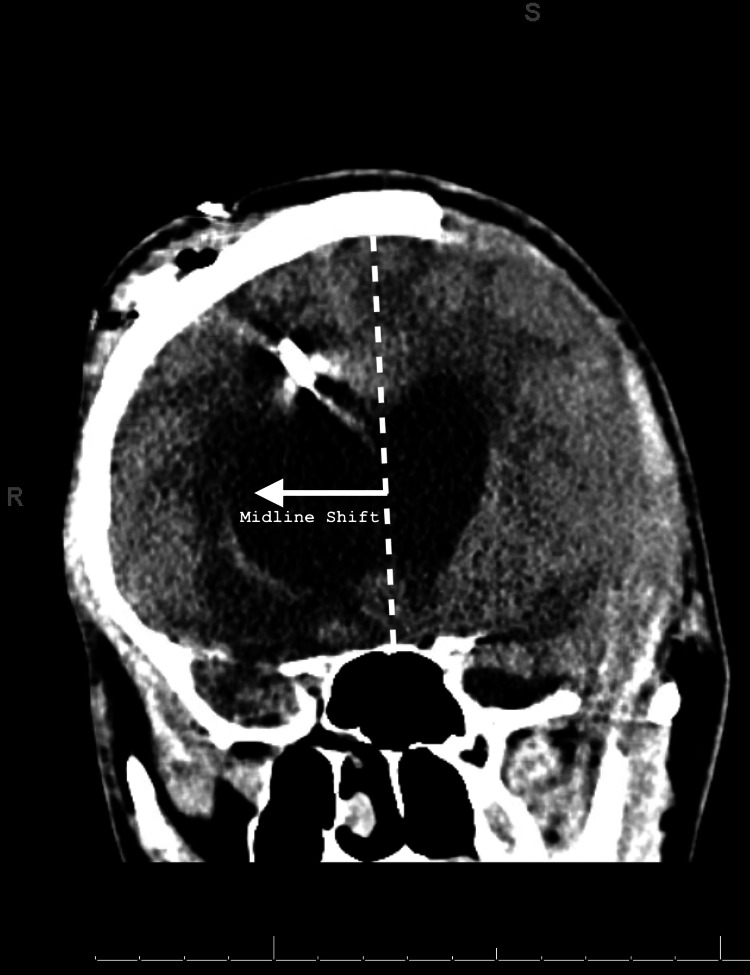
Coronal CT brain demonstrating progressive hydrocephalus with worsening ventricular dilation, diffuse transependymal edema, and marked rightward subfalcine herniation measuring approximately 1.3 cm with midline shift (white arrow), concerning for elevated intracranial pressure secondary to shunt dysfunction. CT: computed tomography

On hospital day 1, the patient underwent shunt evaluation with computed tomography (CT) of the brain and a plain radiographic shunt series. CT imaging of the brain with contrast demonstrated marked ventriculomegaly involving the lateral ventricles with interval worsening, with associated transependymal edema and progressive subfalcine herniation measuring up to 1.3 cm, consistent with elevated intracranial pressure and progressive hydrocephalus (Figures 1-2). Plain radiographic shunt series showed no evidence of mechanical obstruction.

**Figure 3:**
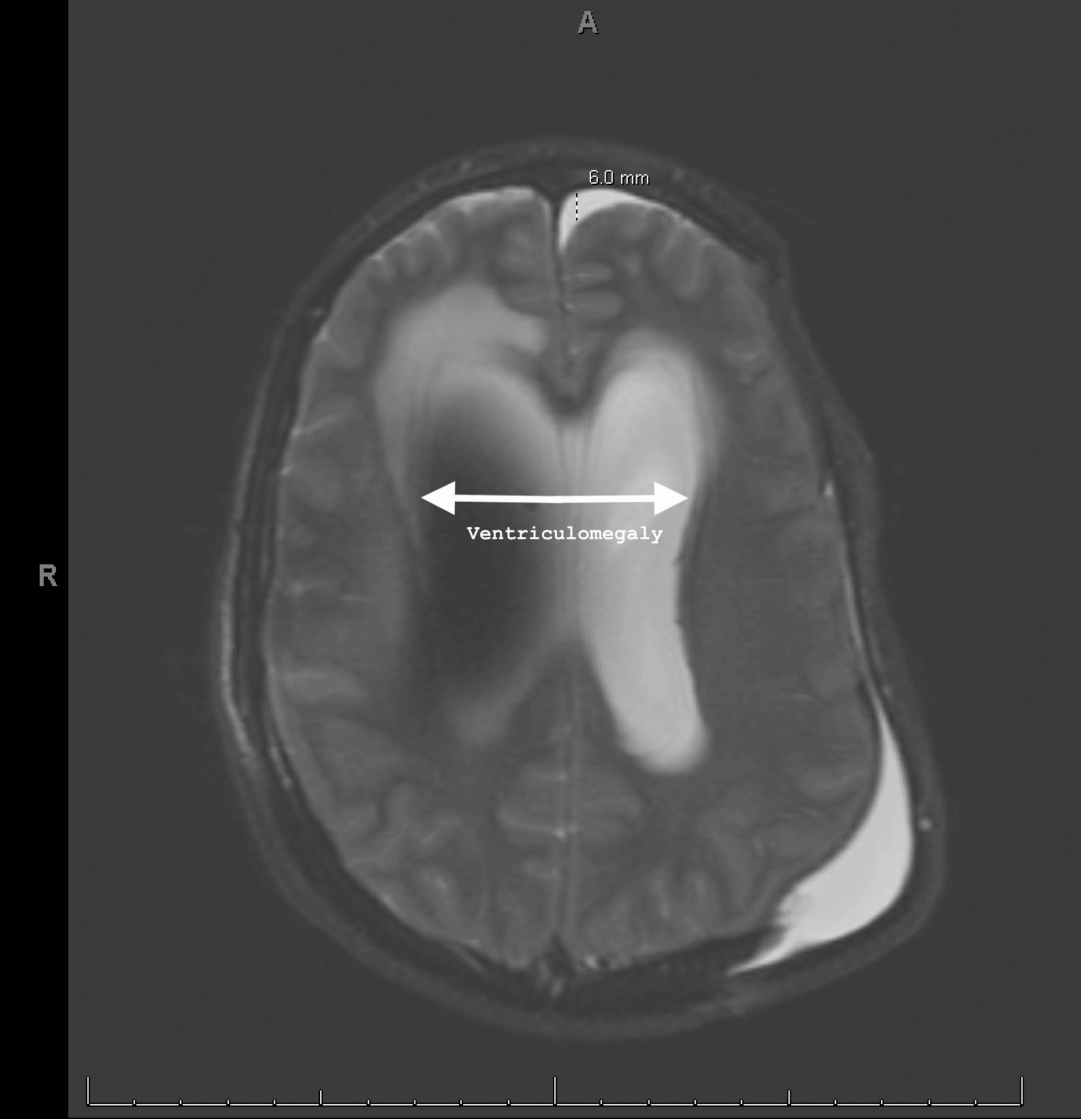
Axial T2-weighted magnetic resonance imaging (MRI) of the brain obtained on hospital day 3 demonstrating persistent severe ventriculomegaly (white arrows) with transependymal edema despite appropriate ventricular catheter positioning, suggesting impaired cerebrospinal fluid diversion.

On hospital day 3, magnetic resonance imaging of the brain demonstrated persistent severe hydrocephalus with transependymal edema despite appropriate positioning of the ventricular catheter, suggesting impaired CSF diversion (Figure 3). No acute infarction was identified.

**Figure 4:**
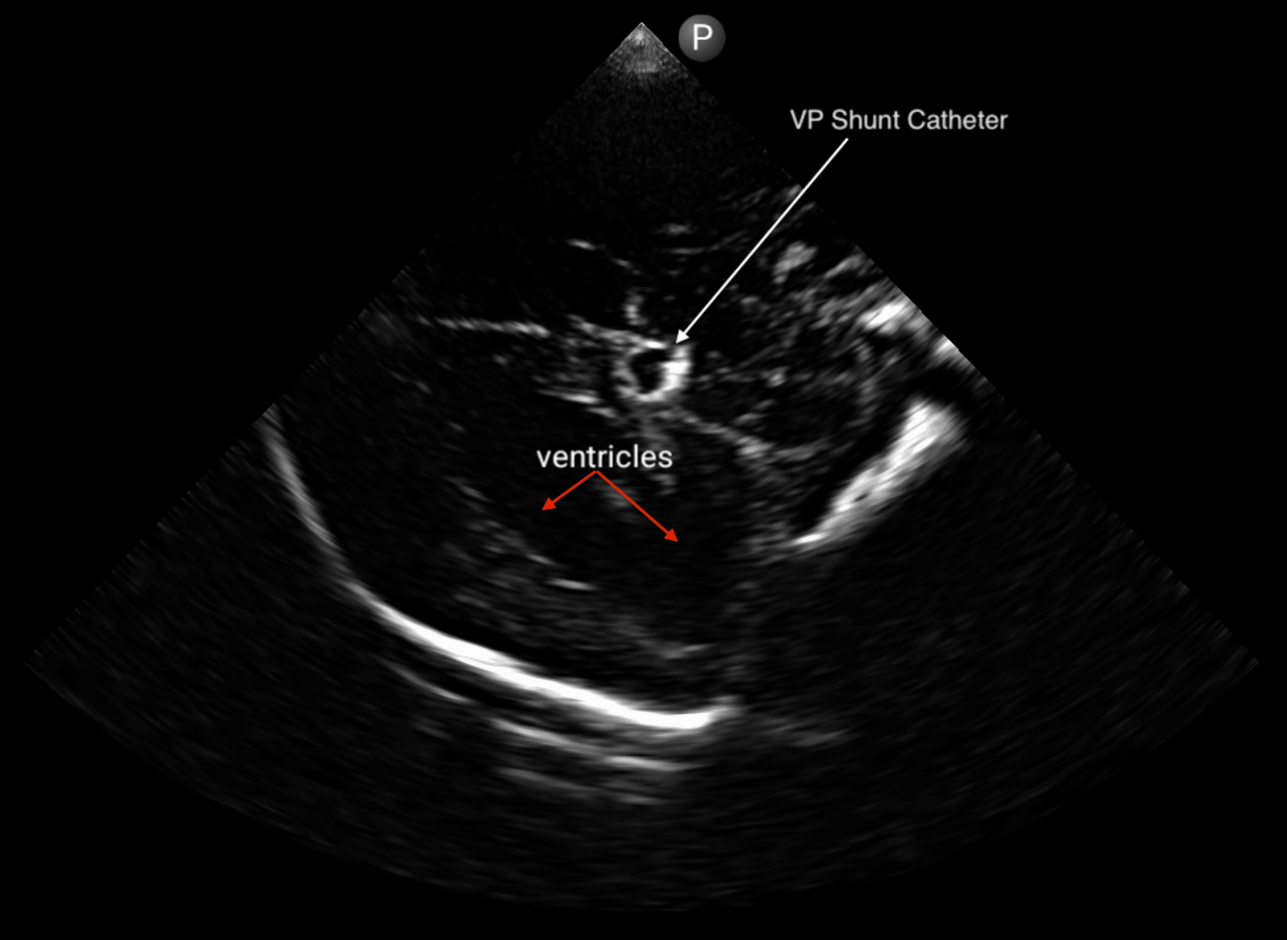
Point-of-care transcranial ultrasound obtained through a previous hemicraniectomy window demonstrating the ventricular system. The ventricles are visualized as anechoic structures (red arrows), allowing direct intracranial assessment. The ventriculoperitoneal shunt catheter is visualized as a hyperechoic linear structure (white arrow) within the ventricular system.

On hospital days 5-8, POCUS was performed due to continued concern from the healthcare proxy regarding possible shunt malfunction. POCUS was performed in the setting of a previous decompressive hemicraniectomy, which allowed direct sonographic visualization to assess VP shunt patency (Figure 4).

**Figure 5:**
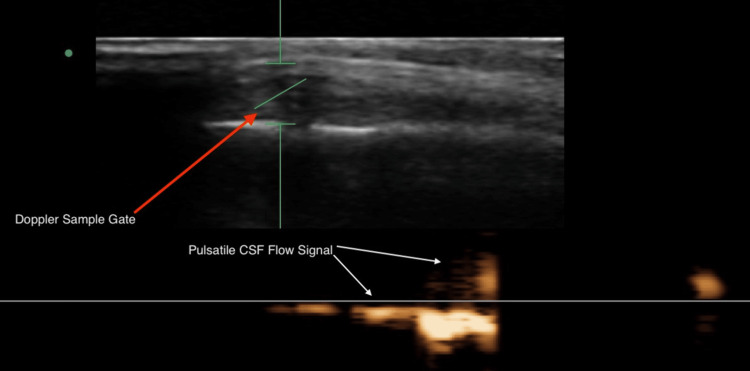
Pulse-wave Doppler ultrasound demonstrating pulsatile cerebrospinal fluid flow (white arrows) within the ventriculoperitoneal shunt. The Doppler sample volume is positioned over the shunt tubing (red arrow), generating a spectral waveform consistent with dynamic CSF flow. CSF: cerebrospinal fluid

POCUS was conducted using the pulse-wave Doppler mode. The ultrasound probe was gently applied along the shunt tract, with brief compression of the distal catheter followed by release to assess the CSF flow. Interrogated sites included the ventricular catheter, shunt reservoir outflow, and the distal catheter segment, which was assessed indirectly by evaluating for free fluid within dependent peritoneal spaces. Anechoic fluid was visualized in the pelvis adjacent to the urinary bladder, consistent with intraperitoneal fluid distribution. While this finding is compatible with distal CSF drainage into the peritoneal cavity, it does not confirm catheter tip location or quantify functional shunt performance. Pulse-wave Doppler interrogation demonstrated characteristic pulsatile flow signals following distal catheter compression and release, demonstrating dynamic CSF movement through the shunt system (Figure 5), consistent with preserved CSF flow within the shunt system (shunt patency), though not necessarily indicative of adequate shunt function. Pulse-wave Doppler interrogation was performed with the sample volume placed over the shunt tubing, with insonation angle minimized to optimize flow detection. Distal catheter compression was applied for approximately 2-3 seconds, followed by release, and the maneuver was repeated to confirm reproducibility. Examinations were performed by clinicians experienced in POCUS. Notably, the patient’s hemicraniectomy allowed direct sonographic visualization of the ventricular system and the proximal shunt catheter, facilitating intracranial assessment of CSF flow (Figure 4). Ultrasound was performed using a Philips Lumify S4-1, 1-4 MHz broadband phased-array transducer (Philips Healthcare; Bothell, WA, USA), allowing adequate penetration for intracranial and abdominal assessment. Pulse-wave Doppler settings were optimized for low-flow detection, including low-pulse repetition frequency and wall filter, with gain adjusted to reduce the background noise. Shunt patency was defined qualitatively by the presence of reproducible pulsatile Doppler waveforms following distal catheter compression and release. Quantitative Doppler flow measurements were not feasible due to technical limitations of bedside assessment, and evaluation was qualitative based on reproducible pulsatile waveforms.

**Figure 6:**
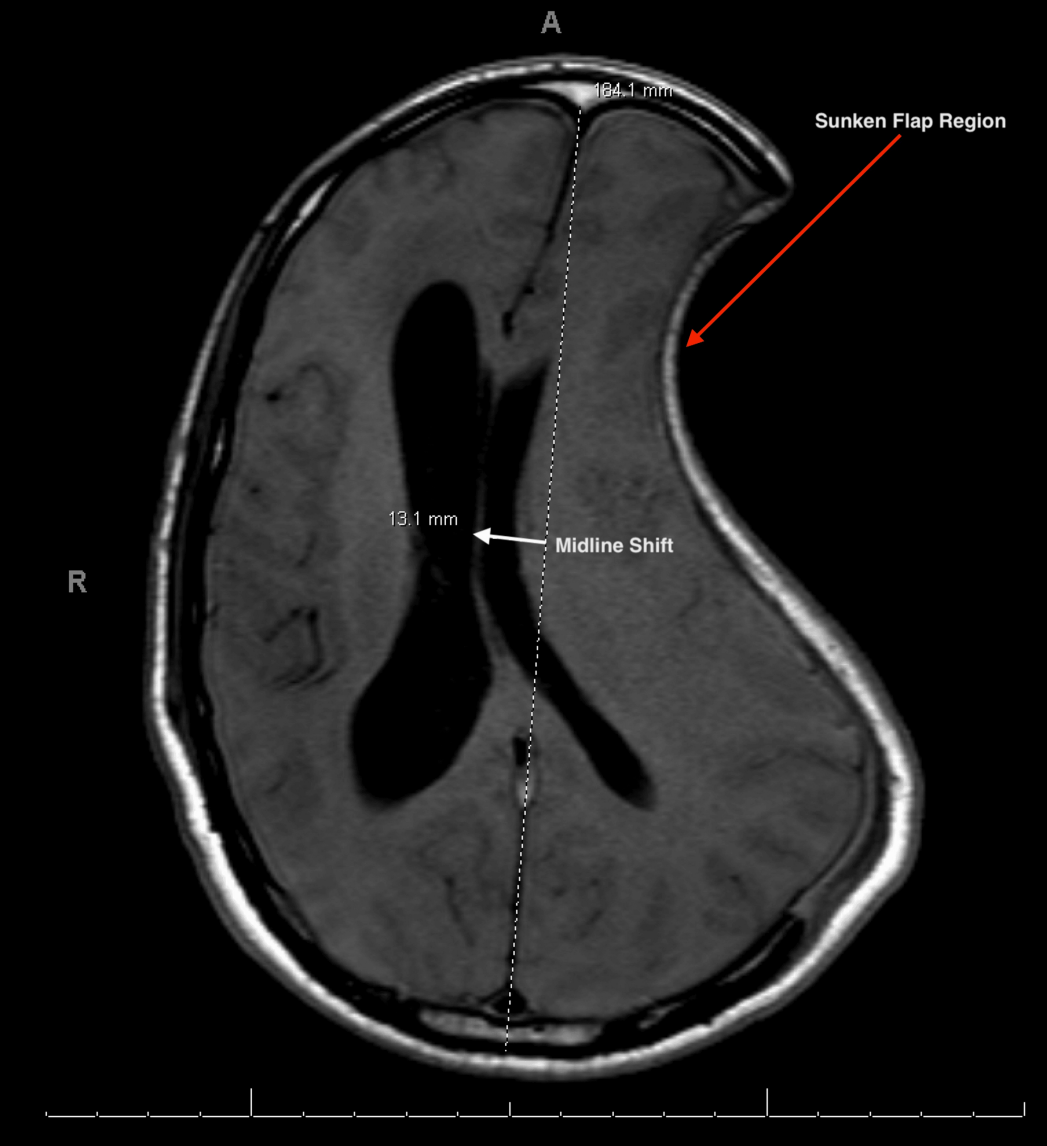
Axial magnetic resonance imaging (MRI) of the brain demonstrating paradoxical brain herniation with approximately 1.3 cm rightward midline shift (white arrow) following decompressive hemicraniectomy, consistent with sinking skin flap syndrome. The sunken flap region is visible along the craniectomy site (red arrow).

On hospital day 17, follow-up magnetic resonance imaging of the brain demonstrated an interval decrease in ventricular size and periventricular edema; however, new findings included paradoxical brain herniation with a 1.3 cm rightward midline shift, effacement of the basal cisterns, and mild uncal herniation, findings consistent with paradoxical brain herniation, or sinking skin flap syndrome (Figure 6).

From hospital days 1 to 3, cross-sectional imaging (Figures 1-3) demonstrated progressive severe hydrocephalus with worsening ventriculomegaly, transependymal edema, and midline shift despite appropriate ventriculoperitoneal shunt positioning. Between hospital days 5 and 8, POCUS (Figures 4-5) demonstrated reproducible pulsatile CSF flow within the shunt system, suggesting preserved shunt patency. Despite these findings, follow-up imaging on hospital day 17 (Figure 6) demonstrated interval reduction in ventricular size with development of paradoxical brain herniation. The patient was managed conservatively with antimicrobial therapy and supportive care, with gradual clinical improvement. A summary of the patient’s clinical course is provided (Table 1).

**Table 1:** Chronological summary of clinical course, imaging findings, and point-of-care ultrasound assessment in suspected ventriculoperitoneal shunt dysfunction POCUS: point-of-care ultrasound; CSF: cerebrospinal fluid; MRI: magnetic resonance imaging; CT: computed tomography; ICP: intracranial pressure; ICU: intensive care unit; VP: ventriculoperitoneal

Hospital day	Clinical status/exam findings	Imaging findings	POCUS findings	Interventions	Clinical course/outcome
Day 1	Presentation with signs of elevated ICP; neurological exam consistent with acute hydrocephalus.	CT brain: severe ventriculomegaly, transependymal edema, 1.3 cm subfalcine herniation. Shunt series: no mechanical obstruction.	-	Urgent neurosurgical and neurology consultation; empiric antibiotic therapy initiated.	Admitted to ICU; hemodynamically monitored.
Day 3	Persistent neurological deficits; no clinical improvement.	MRI brain: persistent severe hydrocephalus; VP shunt catheter in appropriate position.	-	Continued medical management; neurosurgical intervention deferred.	Clinical status unchanged; plan for serial imaging.
Days 5-8	Gradual stabilization; no acute neurological deterioration.	-	Serial POCUS: reproducible pulsatile CSF flow confirmed within VP shunt tubing bilaterally; no flow obstruction identified.	POCUS-guided monitoring continued; no shunt revision warranted.	POCUS findings supported preserved CSF flow within the shunt system.
Day 17	Clinical improvement noted; neurological exam trending toward baseline.	MRI brain: decreased ventricular size; new paradoxical brain herniation with midline shift identified.	-	No additional neurosurgical intervention; antibiotic course continued.	Paradoxical herniation attributed to CSF over-drainage in the setting of infection; managed conservatively.
Discharge/ overall course	Gradual, sustained clinical improvement to near-baseline neurological function.	Interval improvement on serial MRI; ventricular decompression achieved.	POCUS findings supported functional shunt patency throughout hospitalization.	Completed antibiotic course; no surgical revision performed.	Patient discharged with outpatient neurology and neurosurgery follow-up; favorable outcome without operative intervention.

Primary diagnosis

Mechanical ventriculoperitoneal shunt obstruction became less likely following POCUS assessment, demonstrating preserved pulsatile CSF flow within the shunt system.

Final diagnosis

Paradoxical brain herniation (sinking skin flap syndrome) in the setting of decompressive hemicraniectomy with preserved VP shunt patency but impaired intracranial pressure dynamics.

Outcome/progression

In conjunction with reassuring ultrasound findings, no immediate neurosurgical intervention was pursued. The patient continued antimicrobial therapy and supportive care. Over the course of hospitalization, the fever resolved, imaging demonstrated interval improvement in ventricular caliber despite evolving intracranial pressure abnormalities, and neurologic function showed mild improvement.

## Discussion

VP shunt malfunction represents a diagnostic challenge due to its variable and nonspecific clinical presentation. Conventional imaging modalities, while effective, may be limited by access, time constraints, and patient stability [1,2]. Ultrasound-based assessment of shunt patency has been described for several decades, with early work demonstrating the feasibility of sonographic evaluation as a noninvasive diagnostic tool [2].

Subsequent studies have expanded on this concept using Doppler ultrasound techniques to assess CSF flow dynamics. Kaplan et al. identified characteristic CSF flow wave patterns associated with VP shunt obstruction, supporting Doppler ultrasound as a functional assessment tool [1]. More recently, advanced modalities, such as superb microvascular imaging, have further demonstrated the ability to noninvasively visualize shunt patency [3]. Case reports and small series have also highlighted the practical bedside application of POCUS for evaluating VP shunt function when obstruction is suspected [4].

In this case, pulse-wave Doppler POCUS allowed rapid, bedside assessment, suggestive of shunt patency at multiple points along the shunt pathway, despite concerning findings on CT. Detection of free fluid in dependent peritoneal spaces, such as the pelvis, may provide indirect support for distal catheter patency, though these findings are nonspecific and should be interpreted alongside clinical and imaging data. These findings illustrate the complementary role of anatomical and functional ultrasound assessment along the shunt pathway. The presence of a hemicraniectomy uniquely facilitated intracranial sonographic assessment; however, extracranial and distal catheter evaluation techniques remain applicable in patients without cranial defects [1,3,4]. While POCUS should not replace standard neuroimaging or neurosurgical consultation, it may serve as a valuable adjunct, particularly when other diagnostic modalities are delayed, inconclusive, or declined.

Despite promising findings, the current literature remains limited, and further studies are needed to assess reproducibility, sensitivity, specificity, and standardized protocols for ultrasound-based VP shunt assessment [1,3,4]. Operator experience and familiarity with Doppler ultrasound techniques may also influence image acquisition and interpretation, representing an important consideration for broader clinical adoption. 

This case highlights the complex relationship between shunt patency, CSF dynamics, and intracranial pressure physiology. While POCUS demonstrated preserved pulsatile flow within the shunt system, serial imaging revealed persistent hydrocephalus followed by paradoxical brain herniation. These findings indicate that Doppler-detectable flow does not necessarily equate to effective CSF diversion or appropriate intracranial pressure regulation. This case highlights that shunt patency and functional shunt performance are distinct clinical entities that must be interpreted separately. Potential explanations include partial obstruction, valve dysfunction, impaired distal absorption, or altered intracranial compliance in the setting of severe traumatic brain injury.

In this case, the shunt was mechanically patent based on reproducible Doppler-detectable CSF flow; however, it was likely physiologically inadequate for effective intracranial pressure regulation. Potential mechanisms include partial obstruction, valve dysfunction, impaired peritoneal absorption, or altered intracranial compliance following traumatic brain injury and decompressive craniectomy.

The development of paradoxical brain herniation, or sinking skin flap syndrome, represents a condition in which altered cranial mechanics following decompressive craniectomy result in a mismatch between atmospheric and intracranial pressures [5,6]. Importantly, this phenomenon demonstrates that preserved CSF flow and even reductions in ventricular size may not correlate with clinical improvement and may instead reflect pathologic intracranial pressure gradients. 

**Figure 7:**
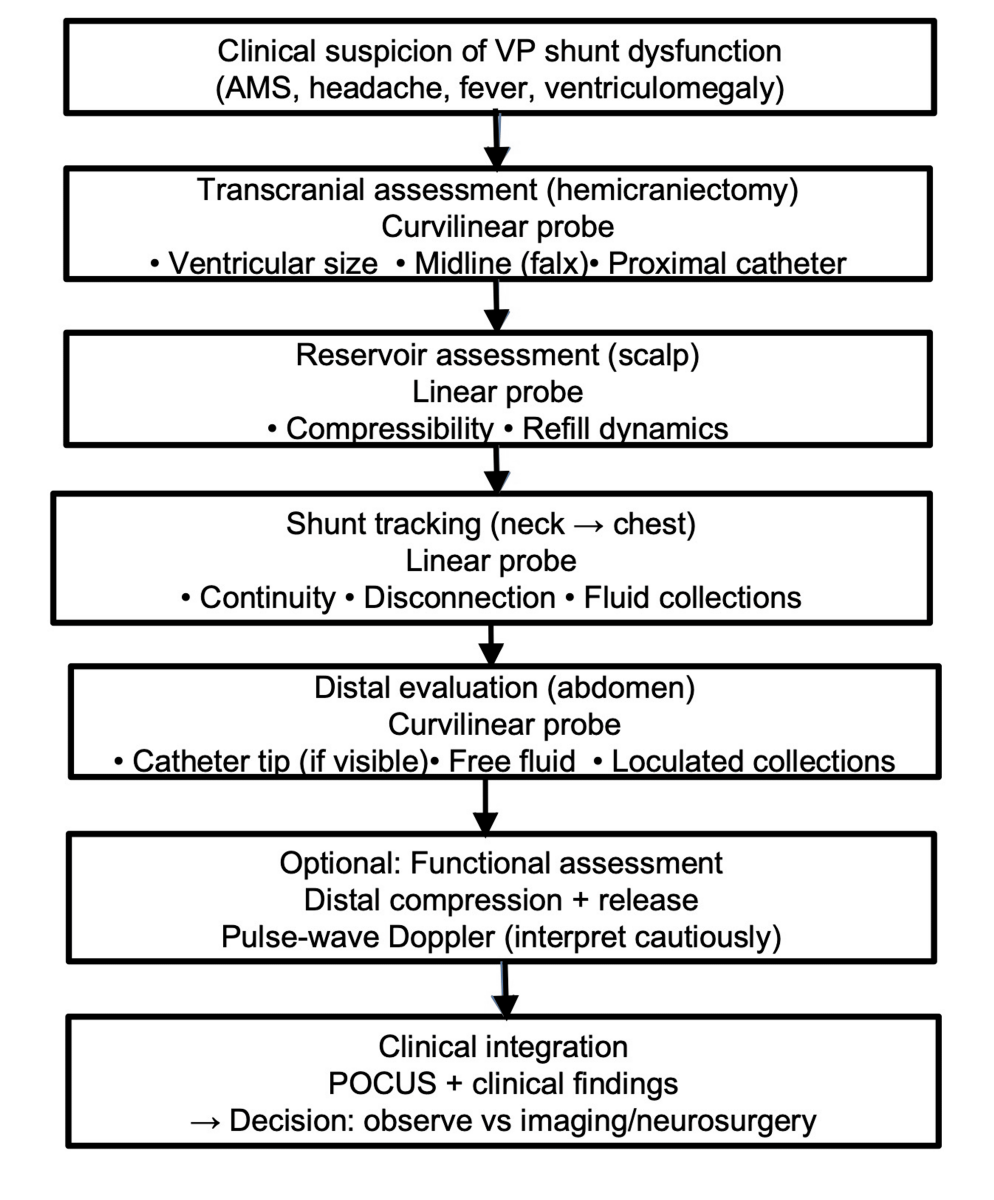
Proposed systematic point-of-care ultrasound algorithm for evaluation of ventriculoperitoneal shunt function in patients with hemicraniectomy Image credit: The image was created by the authors, Joseph A. White and Guillermo Izquierdo-Pretel. VP: ventriculoperitoneal; POCUS: point-of-care ultrasound; AMS: altered mental status

Based on these findings, we propose a systematic bedside POCUS approach that integrates Doppler-based flow assessment with anatomical evaluation, including ventricular size and midline structures, such as the falx cerebri, which may allow estimation of midline shift using sonographic techniques described in neurocritical care literature [6,7]. In patients with hemicraniectomy windows, this may allow the estimation of midline shift and improved bedside monitoring of intracranial dynamics (Figure 7). This structured approach emphasizes that Doppler findings should be interpreted cautiously and always within the broader clinical and radiographic context.

This case has several limitations. The presence of a hemicraniectomy window enabled intracranial visualization, limiting generalizability. Additionally, ultrasound cannot quantify CSF drainage volume or intracranial pressure gradients. The absence of a gold-standard comparator, such as radionuclide shuntography or surgical exploration, limits definitive assessment of shunt function. Additionally, ultrasound assessment is inherently operator-dependent and may be influenced by probe positioning and patient-specific anatomical factors.

## Conclusions

POCUS represents a noninvasive adjunct for rapid evaluation of ventriculoperitoneal shunt patency. Existing literature supports the feasibility of Doppler-based and advanced ultrasound techniques for assessing CSF flow within VP shunts. In patients with suspected shunt malfunction, POCUS may provide timely physiologic information that supports clinical decision-making and reassures both clinicians and caregivers. Critically, however, this case demonstrates that detectable CSF flow (shunt patency) does not necessarily indicate adequate functional shunt performance or normal intracranial pressure dynamics. Integration of ultrasound findings with clinical and radiographic data is essential, particularly in patients with complex intracranial physiology. A multimodal approach incorporating both functional and anatomical assessment may improve bedside evaluation and clinical decision-making. Further prospective studies are needed to validate its diagnostic performance and establish standardized assessment techniques.
